# Modelling of crustal composition and Moho depths and their Implications toward seismogenesis in the Kumaon–Garhwal Himalaya

**DOI:** 10.1038/s41598-021-93469-1

**Published:** 2021-07-07

**Authors:** Prantik Mandal, D. Srinivas, G. Suresh, D. Srinagesh

**Affiliations:** CSIR-National Geophysical Research Institute, Uppal Road, Hyderabad, Telangana 500007 India

**Keywords:** Solid Earth sciences, Seismology

## Abstract

We image the lateral variations in the Moho depths and average crustal composition across the Kumaon–Garhwal (KG) Himalaya, through the H–K stacking of 1400 radial PRFs from 42 three-component broadband stations. The modelled Moho depth, average crustal Vp/Vs, and Poisson’s ratio estimates vary from 28.3 to 52.9 km, 1.59 to 2.13 and 0.17 to 0.36, respectively, in the KG Himalaya. We map three NS to NNE trending transverse zones of significant thinning of mafic crust, which are interspaced by zones of thickening of felsic crust. These mapped transverse zones bend toward the north to form a NE dipping zone of maximum changes in Moho depths, below the region between Munsiari and Vaikrita thrusts. The 1991 M_w_6.6 Uttarakashi and 1999 M_w_6.4 Chamoli earthquakes have occurred on the main Himalayan thrust (MHT), lying just above the mapped zone of maximum changes in Moho depths. Modelled large values of average crustal Vp/Vs (> 1.85) could be attributed to the high fluid (metamorphic fluids) pressure associated with the mid-crustal MHT. Additionally, the serpentinization of the lowermost crust resulted from the continent–continent Himalayan collision process could also contribute to the increase of the average crustal Vp/Vs ratio in the region.

## Introduction

The continent–continent collision between the Indian and Eurasian plates at ~ 55 Ma led to the formation of the Himalayan mountain chain and the Tibetan plateau, the largest continental plateau on the earth. The under-thrusting of Indian plate is still continuing under the Eurasian plane along a north-dipping decollement plane resulting in the occurrences of moderate to great Himalayan earthquakes^1^. The shallow portion of this north-dipping megathrust boundary has been shown to be the nucleation zone for large Himalayan earthquakes^2^. Until today, four great earthquakes exceeding magnitude 8.0 have occurred across the Himalayan frontal arc namely 1505 central Himalaya, 1897 Shillong, 1950 Assam, and 1934 Bihar-Nepal earthquakes^2^. Moreover, the Himalayan region also experienced several earthquakes of M7.5 + (e.g., Kangra, 1905, M7.8, Kashmir, 2005, M7.6, Nepal, 2015, M7.8)^3^. The 1950 Assam event of M_w_8.6 has been recognized as the tenth largest earthquake of the twentieth century. The last large Himalayan earthquake with M_w_7.8 occurred (at 15 km depth) on 25 April 2015 in the region northwest of Kathmandu, and ruptured 100 km toward the east with a maximum recorded displacement of 3 m^4^. Geological and seismic imaging studies in the Himalayas revealed a complicated history of faulting and earthquakes^5–7^. Here we focus on the active main Himalayan thrust (MHT) fault. On MHT, the Indian plate pushes the leading edge of the Eurasian plate northward shortening the overriding crust by over two centimetres per year^1^. On the deeper low friction part of the MHT or plate boundary displacement occurs by slow creep with few earthquakes. In the 15–20 km depth range, frequent magnitude 3 to 6 earthquakes occur on the MHT^1, 2^. The shallow part of the MHT is locked by high friction and stress increases during motion on the fault at deeper levels^8^. Other major Himalayan thrusts (like MCT, MBT and MFT) root into the MHT, which has been mapped through different seismological, geophysical and geological studies. The tomographic imaging of the rupture zone of the 2015 Nepal earthquake showed that the MHT is a low velocity zone between 15 and 30 km depths^7^. Several studies have been carried out to model the crust-mantle boundary in the Himalayas. These studies have shown a significant lateral variation of Moho depths in different parts of the Himalaya. In Kumaon–Garhwal (KG) Himalaya, the Moho depths are modelled to 35–45 km in lower Himalaya and 50–55 km in Higher Himalaya while they vary between 40 and 70 km in Nepal and Tibet^9^, and 35–50 km in the north-eastern Himalaya^10^. A 65-km thick crust in Tibet has also been modelled by gravity data^11^. However, 3-D mapping of the MHT and Moho depths remains to be crucial for imaging the extent of ruptures for large future earthquakes.

**Figure 1:**
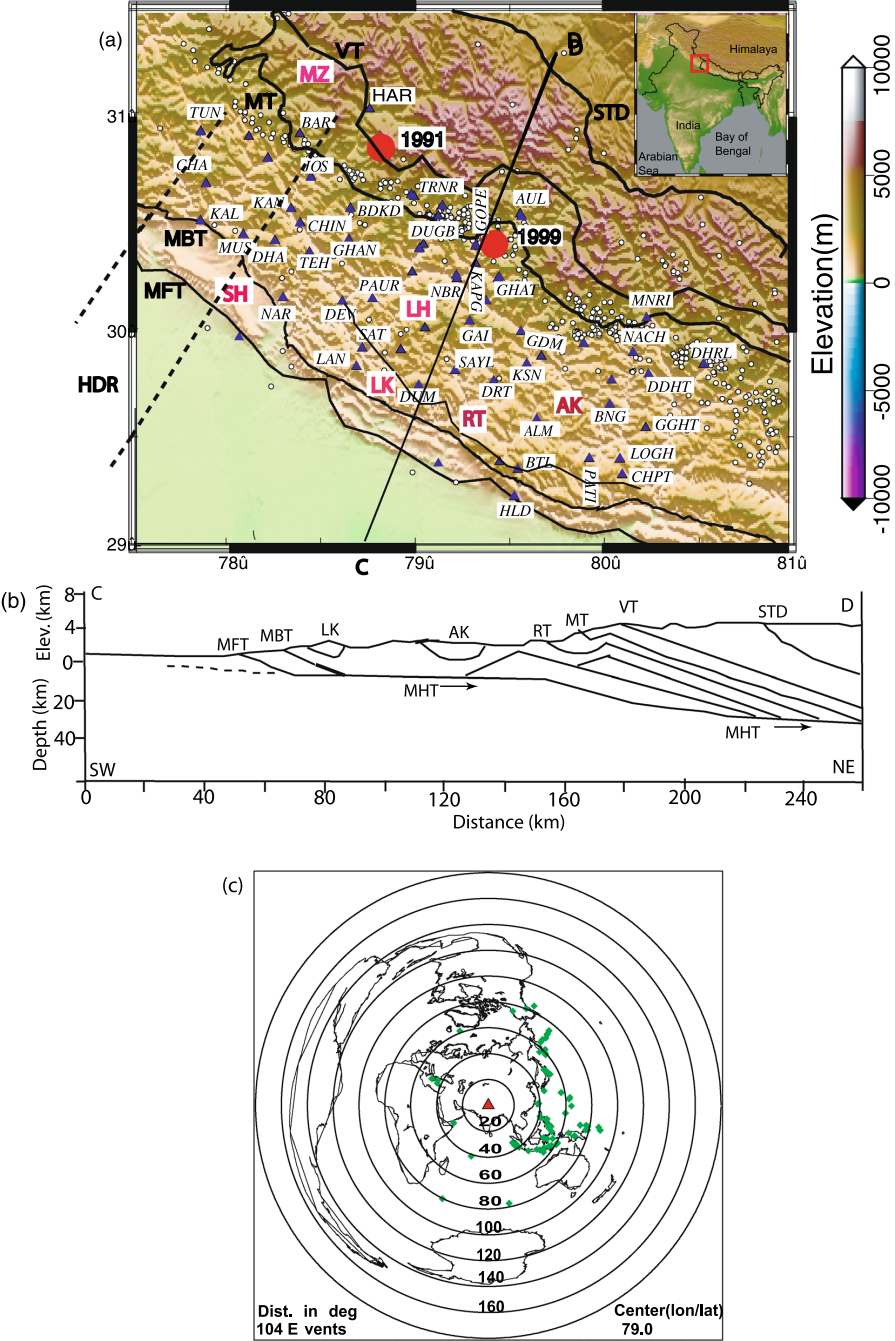
(**a**) Station location map of the Kumaon—Garhwal (KG) Himalayan region. Filled violet triangles (with abbreviated names) mark the location of broadband stations while small filled white circles mark the earthquake relocations obtained from simultaneous inversion. Two large filled red circles mark the epicentral locations of the 1991 Uttarkashi and 1999 Chamoli earthquakes. The solid black line represents major faults. *MT* Munsiari thrust, *VT* Vaikrita thrust, *MBT* Main Boundary Thrust, *MFT* Main Frontal Thrust, *RT* Ramgarh Thrust, *MHT* Main Himalayan Thrust. SH, LH, AK, LK and MZ mark Siwalik Himalaya, Lesser Himalaya, Almore klippe, Lansdown Klippe and MCT zone, respectively. Figure 1a is generated using the Generic Mapping Tool (GMT) software version 6^41^ (https://doi.org/10.1029/2019GC008515). Black dotted lines mark the NE extension of the Delhi – Haridwar ridge (HDR)^2^. The elevation data used in generating GMT plot is obtained from the open source Digital Elevation Model (DEM) (https://asterweb.jpl.nasa.gov/gdem.asp), (**b**) Tectonic depth cross-section^14^ across the NE–SW CD profile, whose location is shown in **a**, and (**c**) Epicentral plot of 104 teleseismic events, whose broadband data from the Uttarakhand network, are used for our P-receiver function study. A red triangle and green diamond symbols mark the center of our network (Lat. 79°, Long. 30°) and epicenters of selected teleseismic events.

The central Himalayan GAP area, which lies between the rupture zones of the 1905 Kangra and 1934 Bihar-Nepal main events, to the west of the 2015 M_w_7.8 Nepal earthquake had witnessed several large M ≥ 6 interplate earthquakes at various places, e.g., 1344 Garhwal (M ≥ 8), 1505 Lo Mustang (M ~ 8.2), 1803 Garhwal (M ~ 7.7), 1905 Kangra (M7.8), 1975 Kinnaur (M6.8), 1991 Uttarkashi (M6.8) and 1999 Chamoli (M6.4)^12^ (Fig. 1a). A geological cross-section of the Kumaon Himalaya showing MHT has been constructed by Srivastava and Mitra^6^ (Fig. 1b). The lower flat portion of the MHT has been shown as the nucleation zones for the 1991 Uttarkashi, M_w_6.8 and 1999 Chamoli, M_w_ 6.4 earthquakes^12^. However, this GAP area has not experienced any large earthquake during the last 500 years, thus, it is imperative to study the seismicity vis-â-vis the crustal structure for understanding the seismo-tectonics and the seismic hazard associated with the Uttarakhand region in the central Himalaya. Furthermore, the recent GPS study suggested a long-term convergence rate of 18 mm/yr, which along with strongly coupled MHT for > 500 years makes this region highly vulnerable zone for the occurrence of large future earthquakes^13^. To understand the seismogenesis, CSIR-National Geophysical Research Institute (CSIR-NGRI), Hyderabad, has been operating a regional network of 56 three-component broadband stations in the Uttarakhand Himalayan region (Fig. 1a), since 2017, which provided us a dataset of 1400 good teleseismic earthquakes (Fig. 1c) to carry out a detailed 3-D mapping of the MHT and Moho in the KG Himalaya, through inversion of P-RFs. In this paper, we present the modelled average crustal Vp/Vs ratios and Moho depths at 42 stations, which in turn provide the 3-D spatial distribution of Moho depths and crustal composition, in the Uttarakhand Himalaya.

Four principal geological subdivisions namely Siwalik Himalaya (SH), Lesser Himalaya (LH), Higher Himalaya (HH), and South Tibetan detachment (STD) characterize the Kumaon–Garhwal Himalayas^14^. Based on geology, the Lesser Himalaya is further sub-divided into two parts viz., inner Lesser Himalaya (ILH) and outer Lesser Himalaya (OLH) (Fig. 1a,b). While four major thrust fault systems namely Himalayan frontal thrust (MFT), main Boundary thrust (MBT), main Central thrust (MCT), and South Tibetan detachment (STD) define the tectonics of the region^6^. The boundary between SH and Indo-Gangetic plain is marked by MFT while LH is limited by MBT in the south and MCT in the north (Fig. 1a,b). The boundary between HH and LH is also defined by the MCT while the HH is separated from the ITSZ by the STD. Here, the MCT zone (MZ) is observed to be consisting of two faults namely Munsiari (MT) and Vaikrita (VT) (Fig. 1a)^14^. The main link fault for all major thrusts including many local faults in the region defines a low angle north dipping plane, separating the under-thrusting Indian plate from the overriding Eurasian plate. This detachment plane is named as the main Himalayan thrust (MHT), on which most of the major Himalayan earthquakes have occurred. Besides, some NE-striking transverse basement ridges (like the Delhi-Haridwar ridge (HDR), the Faizabad ridge (FZR), and the Munger-Saharsa ridge) in the Ganga basin have been reported^15^. The DHR has been inferred to be extended below the Higher Himalaya, resulting in the creation of Tibetan graben^15^.

## Modelling of P-receiver functions

**Figure 2:**
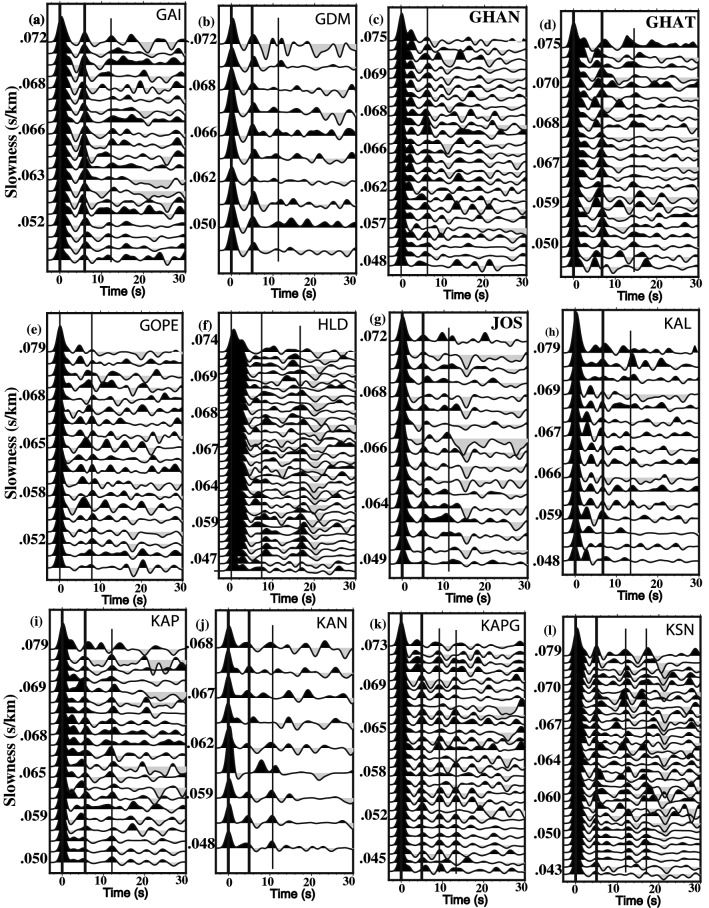
Plots of individual RFs as a function of the horizontal slowness after distance moveout correction for the *Ps* phase to a reference distance of 67^◦^ and slowness 6.4 s deg^−1^, for 15 broadband sites in the KG Himalaya, (**a**) GAI, (**b**) GDM, (**c**) GHAN, (**d**) GHAT, (**e**) GOPE, (**f**) HLD, (**g**) JOS, (**h**) KAL, (**i**) KAP, (**j**) KAN, (**k**) KAPG, and (**l**) KSN. The PRFs at each station show strong azimuthal variation. The conversions from Moho and crustal multiples are marked by solid black lines.

The adequate coverage of broadband stations in the Uttarakhand Himalaya has been possible for the first time by the deployment of 42 mobile 3-component broadband seismographs in October 2017, which enabled us to study the nature of Moho and average *Vp/Vs* ratio^16^, beneath the individual seismograph location in the region. In the present study, for estimating PRFs, we used the best quality digital waveforms of 104 good teleseismic earthquakes of magnitudes ≥ 5.5, with high signal-to-noise ratio and clear P-arrivals (with back azimuth between 38° and 300°, epicenters between 30°S and 90°N, and ray parameters ranging from 0.040 to 0.080 s/km) (Figs. 1c and S1). First, the two horizontal components of seismograms are rotated using the back-azimuths to determine the radial and transverse components of the seismograms, which are then used to estimate the radial and transverse RFs using the iterative time domain deconvolution procedure of Ligorria and Ammon^17^ with 200 iterations. In the time domain deconvolution, the frequency content of the RF is controlled by the Gaussian filter parameter 'a'. Here, a Gaussian width factor, a = 2.5 (f < 1.25 Hz), is considered for estimating high frequency RFs for each event, which provides better recognition of Moho conversion and multiples. Finally, those deconvolutions that reproduced less than 90% of the signal energy on the radial component (when convolved back with the vertical trace) are discarded. Here, we use 1500 radial P-receiver functions showing clear P-to-s conversions associated with the Moho and other crustal multiples (Fig. S1), from 42 broadband stations in the Uttarakhand region (Fig. 1a), to conduct the H–K stacking of P-RFs (Fig. 2a–l, see Supplementary Figs. S1-5).

After obtaining the move-out corrected radial receiver functions for different stations (using Eqs. 2–4), the average crustal Vp/Vs and Moho depth (z_M_) were estimated using the approach of Zhu and Kanamori^16^. In this technique, a grid search over the Vp/Vs- z_M_ space is performed with a view to obtain the (Vp/Vs, z_M_) pair, which is the closest agreement with observed P_s_, PpP_m_s and PsPms + PpSms phases. It is observed that these phases are quite clear on the estimated radial RFs for almost all the stations (Figs. 2a–l, S2a-0, and S3a-j).

The moveout times are a function of the P wave slowness (i.e., the distance), the crustal thickness, and the Vp/Vs ratio. They only weakly depend on the absolute average crustal P wave velocity^16^. In this method, the arrival times of Ps, PpPms and PpSms + PsPms phases are predicted using Eqs. (2–4) and weighting factors w_1_, w_2_, and w_3_ in the Eq. (1) are chosen to balance the contribution from different phases as mentioned above. Using radial PRFs from events at different distances, and stacking all the appropriately shifted traces, gives a robust estimate for the thickness of the crust and (with larger uncertainty) for the Vp/Vs ratio. In our analysis, we set w_1_ = 0.7, w_2_ = 0.2 and w_3_ = 0.1. This combination of weighting factors provides good estimates of H and k for most of the stations. But for some stations, we have got better results for w_1_ = 0.5, w_2_ = 0.3, and w_3_ = 0.2 while some stations gave better results for w_1_ = 0.34, w_2_ = 0.33, and w_3_ = 0.33. For our H–K stacking study, we vary H values from 20 to 70 km and k values from 1.4 to 2.2. Finally we have considered those measurements of (H, k), which show a clear closure between H and k.

**Figure 3:**
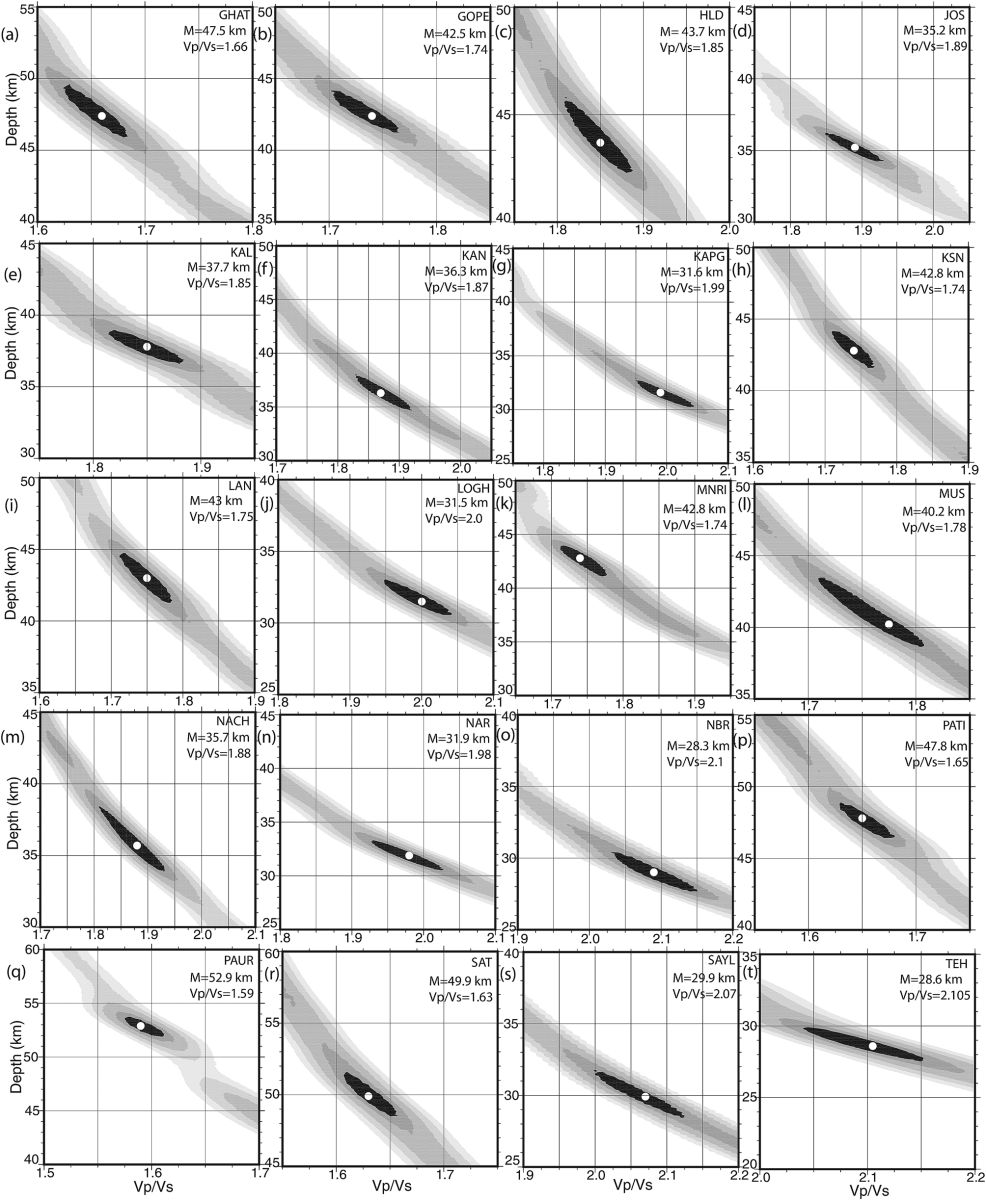
H–K Stacking of PRFs at 20 broadband seismograph sites in the KG Himalaya: (**a**) GHAT, (**b**) GOPE, (**c**) HLD, (**d**) JOS, (**e**) KAL, (**f**) KAN, (**g**) KAPG, (**h**) KSN, (**i**) LAN, (**j**) LOGH, (**k**) MNRI, (**l**) MUS, (**m**) NACH, (**n**) NAR, (**o**) NBR, (**p**) PATI, (**q**) PAUR, (**r**) SAT, (**s**) SAYL, and (**t**) TEH. The best estimated H and K values are indicated by a small white filled circle at the centre of the black error ellipse.

**Figure 4:**
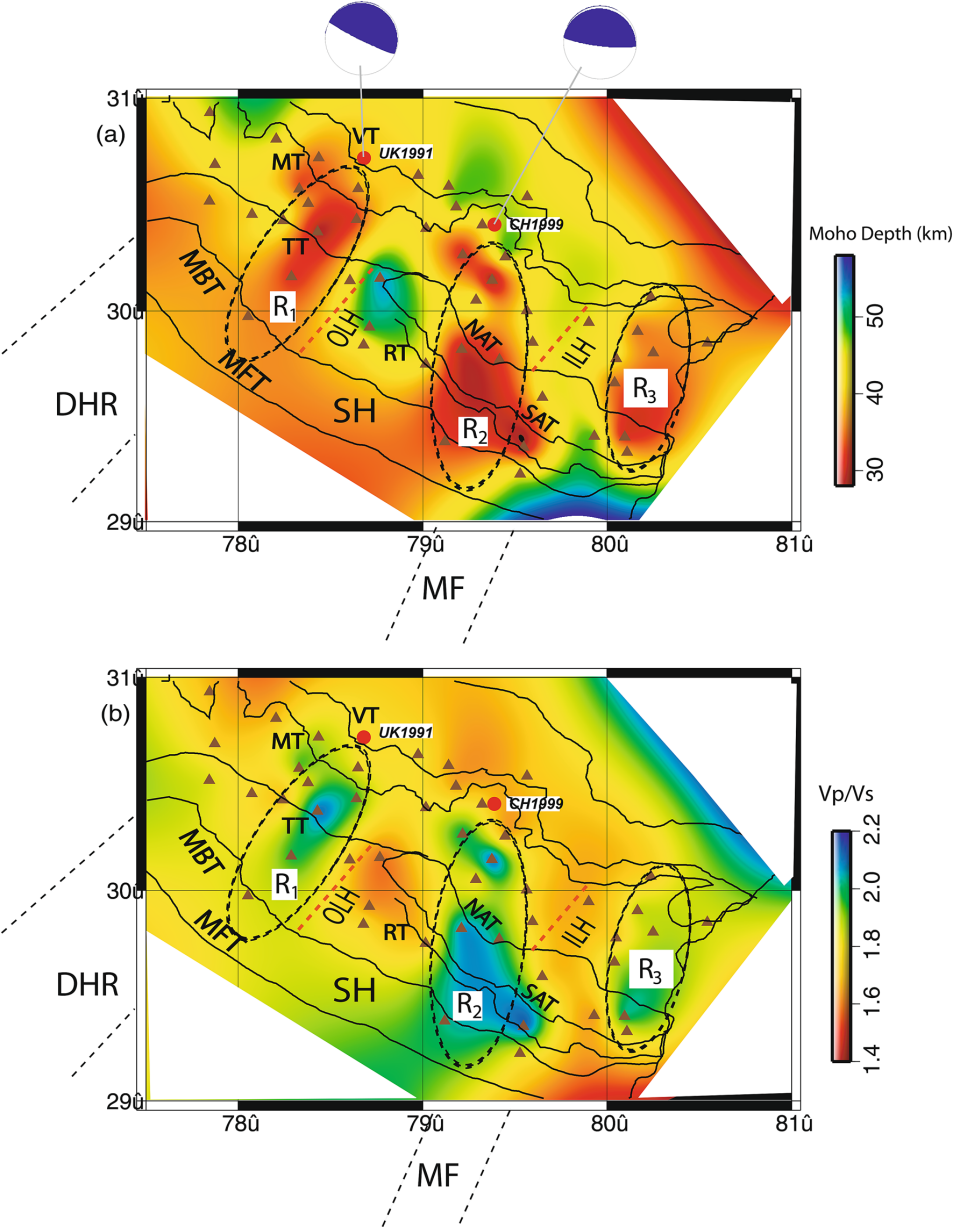
Modelled lateral variations of (**a**) Moho (in km. Black filled triangles show broadband seismic stations. The locations of the 1991 Uttarkashi (M_w_6.6) and 1999 Chamoli (M_w_6.4) earthquakes are shown by large filled red circles. The focal mechanism solutions of these two earthquakes are also shown by beach balls. Solid grey circles mark relocations of local Himalayan events, and (**b**) average crustal Vp/Vs. DHR marks the inferred NE extension of the Delhi-Haridwar basement ridge while MF represents a NE striking inferred Moradabad fault^23^. Black dotted elliptical zones mark the mapper transverse features (N to NE trending). Major thrusts (shown by black lines): *VT* Vaikrita Thrust, *MT* Munsiari Thrust, *TT* Ton Thrust, *RT* Ramgarh Thrust, *NAT* North Almora Thrust, *SAT* South Almora Thrust, *MBT* Main Boundary Thrust, *MFT* Main Frontal Thrust. SH marks the Siwalik Himalaya. ILH marks the inner lesser Himalaya while OLH marks the outer lesser Himalaya.

**Figure 5:**
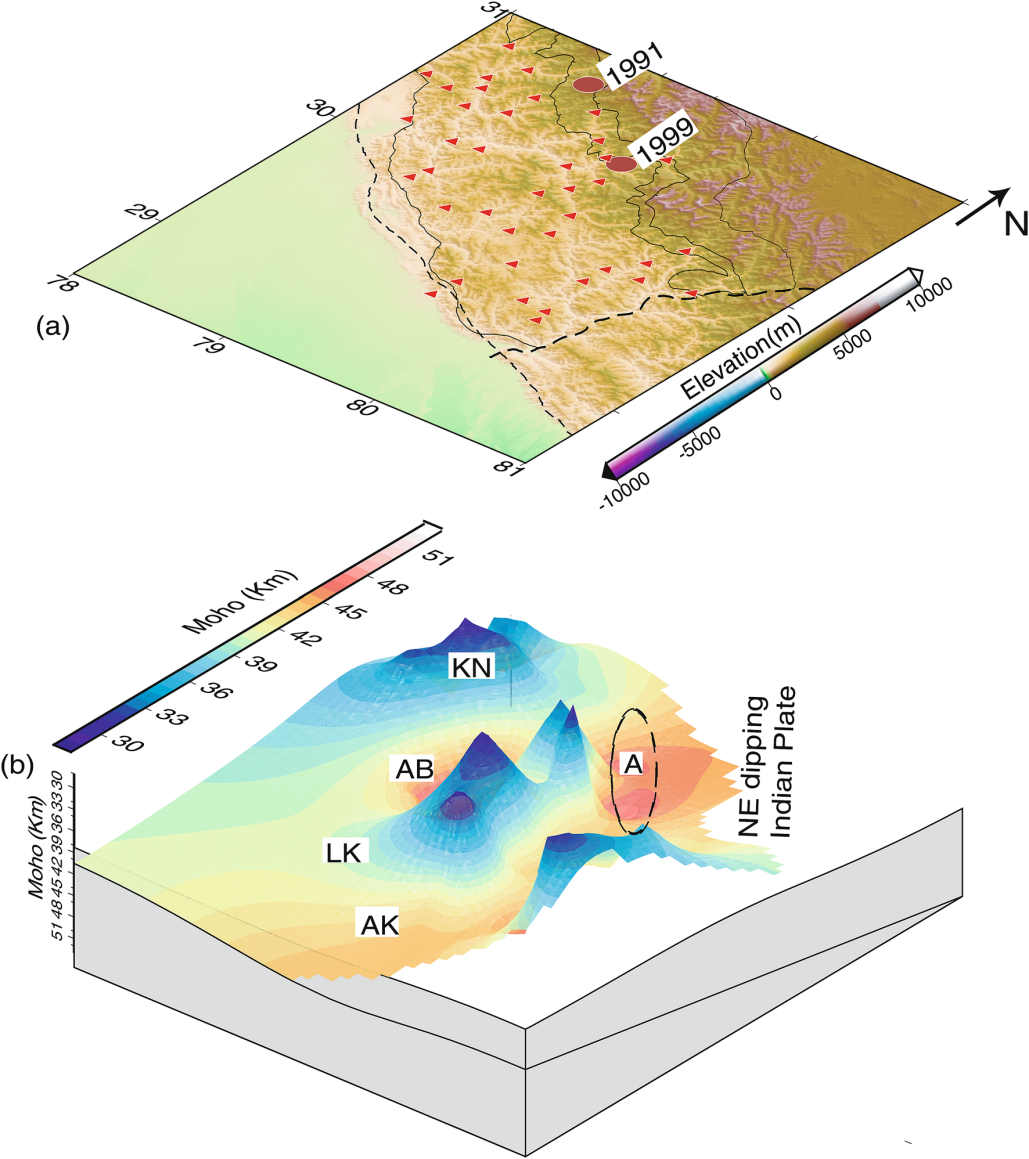
(**a**) Elevation (in m) map along with seismic stations (marked by red filled triangles). While two large red filled circles represent the locations of the 1991 M_w_6.6 Uttarkashi and 1999 M_w_6.4 Chamoli earthquakes. 3-D surface plots of modelled (**b**) Moho (km). The zone-A where maximum changes in Moho depths are modelled while “NE dipping Indian plate” marks the inferred north-easterly subducted Indian Moho. And, AK, LK, AB and KN mark the approximate locations of Almora Klippe, Lansdowne Klippe, Alkananda basin and Kroll Nappe, respectively.

The same procedure of HK stacking of 1500 radial PRFs is performed to estimate the H and k for all 42 broadband stations in the Kumaon—Garhwal Himalaya (Figs. 2a–l, 3a–t, see Supplementary Figs. S2-5). The contours of our estimated Moho depths (in km) and average crustal Vp/Vs values are shown in Fig. 4a,b. A 3-D structural model for the Kumaon–Garhwal Himalaya based on our modelling results is also proposed in Fig. 5a,b, which suggest occurrence of most of the micro-seismicity in the regions, which are characterized by a clear crustal thinning and large Vp/Vs values (i.e. mafic crustal composition). Modelled Moho depths, Vp/Vs and Poisson ratios (from Eq. 7) at 42 broadband stations are listed in Table 1.

**Table 1:** Modelled Moho depths and average crustal Vp/Vs at 42 broadband stations in the Uttarakhand Himalaya.

S. No	Station	Long. (^o^E)	Lat. (^o^N)	Moho depths (km)	Vp/Vs	Poisson’s ratio
1	ALM	79.65	29.59	42.5	1.75	0.26
2	AUL	79.57	30.54	45.5	1.70	0.24
3	BAR	78.21	30.81	45.5	1.69	0.23
4	BDKD	78.65	30.58	38	1.82	0.28
5	BNG	80.05	29.78	42	1.73	0.25
6	BTL	79.55	29.36	28.3	2.13	0.36
7	CHA	77.87	30.69	43.0	1.73	0.25
8	CHIN	78.33	30.58	33.5	1.95	0.32
9	CHPT	80.11	29.33	37.5	1.86	0.30
10	DDHT	80.25	29.80	35.5	1.89	0.31
11	DEV	78.61	30.14	43	1.72	0.25
12	DHA	78.24	30.43	44	1.71	0.24
13	DHRL	80.54	29.85	37.5	1.87	0.30
14	DRT	79.42	29.77	32	1.99	0.33
15	DUGB	79.18	30.49	47.5	1.65	0.21
16	DUM	79.02	29.75	42.5	1.74	0.25
17	GAI	79.29	30.05	43.5	1.83	0.29
18	GDM	79.56	30.00	42	1.78	0.27
19	GGHT	80.04	29.66	35.5	1.87	0.30
20	GHAN	78.64	30.43	32	1.97	0.33
21	GHAT	79.45	30.26	47.5	1.66	0.22
22	GOPE	79.32	30.41	42.5	1.74	0.25
23	HLD	79.53	29.23	43.7	1.85	0.29
24	JOS	78.44	30.72	35.2	1.89	0.31
25	KAL	77.84	30.52	37.7	1.85	0.29
26	KAN	78.38	30.51	36.3	1.87	0.30
27	KAPG	79.22	30.27	31.6	1.99	0.33
28	KSN	79.60	29.85	42.8	1.74	0.25
29	LAN	78.68	29.84	43	1.75	0.26
30	LOGH	80.09	29.40	31.5	2.0	0.33
31	MNRI	80.24	30.07	42.8	1.74	0.25
32	MUS	78.07	30.46	40.2	1.78	0.27
33	NACH	80.17	29.91	35.7	1.88	0.30
34	NAR	78.29	30.16	31.9	1.98	0.33
35	NBR	79.38	30.15	28.3	2.1	0.35
36	PATI	79.93	29.41	47.8	1.65	0.21
37	PAUR	78.77	30.16	52.9	1.59	0.17
38	SAT	78.71	29.92	49.9	1.63	0.20
39	SAYL	79.21	29.82	29.9	2.07	0.35
40	TEH	78.43	30.37	28.6	2.105	0.35
41	TRNR	78.98	30.64	39.7	1.78	0.27
42	TUN	77.85	30.93	46.1	1.68	0.23

Our present study using data from 42 stations has enabled us to obtain a good and robust spatial distribution of Moho depths and average crustal Vp/Vs ratio values in the Kumaon–Garhwal Himalaya. This offers a unique opportunity to explore the relationship between geographical coverage and nature of crustal composition/Moho depths in the region. Further, the network density and geometry enable us to characterize the study region in different zones of tectonic importance. Understanding how the nature of crustal composition and Moho depths throughout the network is critical for the understanding of the crustal evolution in the Kumaon–Garhwal Himalaya. Furthermore, here we combine existing information from different geological and geophysical studies to validate our interpretations. While earlier receiver function studies in the region using with limited numbers of stations (13–20 stations in seismic array mode and 8–10 stations in network mode) in the region have allowed to study crustal composition and Moho depths in different parts of the Kumaon–Garhwal Himalaya, thus, these studies could not obtain a model for crustal evolution for the whole region^18–23^. Thus, our modelling provides new and robust estimates of Moho depths and average crustal Vp/Vs ratio values in the Kumaon–Garhwal Himalaya, which enabled us to derive some new inferences related to the crustal structure and composition below the region lying in the central Himalayan GAP area that has the maximum probability of occurrences of a large to great size earthquakes in future.

## Results and discussions

The H–K stacking of radial P-receiver functions at 42 individual broadband locations (Table 1) are presented in this paper, which reveals marked lateral variations in the crustal composition and Moho depths. The modelled crustal thickness varies from 28.3 km (at BTL) to 52.9 km (at PAUR) in the Kumon–Garhwal Himalaya (Table 1; Fig. 3, see Supplementary Figs. S4,5). The thinnest crust of 28.3 km thickness is obtained at BTL (Table 1), which is less than the estimates of Moho depths (~ 35–45 km) in the northwest Himalaya^18^ and Garhwal Lesser Himalaya^5^. However, the thickest crust of 52.9 km is noticed at PAUR (Table 1), which falls in a zone of thickening of crust, with an average crustal thickness of 45.18 km (i.e. mean of the modelled Moho depths at DEV, SAT, LAN, and DUM) (Table 1; Fig. 3, see Supplementary Figs. S4, 5). We also noticed a zone of crustal thickening just north of MT occupying BAR, TRNR, DUGB, AUL and MNRI sites (Table 1; Fig. 3, see Supplementary Figs. S4, 5). We infer that our modelled Moho depths at 42 stations in Uttarakhand are in good agreement with the results from other seismological and magneto telluric studies in northwest Himalaya^5, 18–26^. We detect two zones of marked changes in Moho depths with large average crustal Vp/Vs values associated with the hypocentral locations of the 1991 UK and 1999 Chamoli events (Fig. 4a,b). These figures also show a zone with marked Moho up-warping and larger crustal Vp/Vs values, below the region just south of the MT, where most of the micro-earthquakes are observed to occur. Thus, we can infer that the regions below the MCT zone, which are characterized by marked up-warping of the mafic crust (Fig. 5b), could be probable future locales of moderate to large earthquakes in the Kumaon–Garhwal Himalayas. This scenario changes as we move toward the south away from the MT. We also notice some important geological features like Almora and Lansdowne klippes are characterized by Moho thickening of relatively felsic crust while Kroll nappe is found to be characterized by marked thinning of mafic crust (Fig. 4a,b). We observe a clear thickening of felsic crust below the Alkananda basin (i.e. AB as shown in Fig. 5b). Two distinct transverse zones (viz., R1 and R2 as shown in Fig. 4a,b) with marked thinning of mafic crust are modelled on the west and SE side of the study region. The R1 and R2 zones spatially correlate well with the inferred locations of the NE-ward extensions of Delhi-Haridwar ridge (DHR) and Moradabad fault (MF), respectively^27^. These transverse features DHR and MF must have intruded already existing Indian crust which has gone under the upper crust of the Eurasian plate. Thus, after the subduction these features could be considered as the lower crustal intrusion below the MHT. Thus, these observations at Moho could be interpreted as a lower crustal intrusion layer (may be due to the northward extension of DHR/MF) at the base of a mafic crust^28^. The northern ends of these two mapped crustal features (viz., R1 and R2) bend below the MCT zone and the region just north of it, probably representing the northeast-ward subducted Indian plate. Our modelling also detects the zone-A (Fig. 5b) where maximum changes in Moho depths are modelled. Interestingly, the vertical downward projections of the hypocenters of both 1991 Uttarkashi (UK) and 1999 Chamoli (CH) earthquakes are lying within this zone A. Thus, the occurrences of the 1991 M_w_6.6 UK and 1999 M_w_6.4 CH thrust earthquakes could be attributed to the large stress accumulation due to the marked changes in Moho depths (i.e. the northward bending of the Indian plate) below the MCT zone and the continued northward under-thrusting of the Indian plate at a rate of ~ 14 mm/year^13^. All earlier studies have proposed that most of the large Himalayan earthquakes took place on seismogeneic down-dip part of the mid-crustal ramp associated with the MHT where most of the strain accumulates due to the convergence of Indian plate^1, 2^. Our 3-D structural model (Fig. 5b) maps a clear north-easterly dipping surface with a marked thinning of Indian mafic crust, probably representing the subducted Indian plate.

The spatial distribution of modelled crustal Vp/Vs ratios (Fig. 4b) reveals a marked lateral variation in the crustal composition below the Uttrakhand Himalaya. The modelled average crustal Vp/Vs, and Poisson’s ratio estimates vary from 1.59 to 2.13 and 0.17 to 0.36, respectively. The regions with high Vp/Vs values (> 2.1) in the Japanese and Cascadian subduction zones have been interpreted as the zones of high fluid pressure^29–31^. Alternatively, serpentinites can also be resulting in anomalously high Vp/Vs ratio and Poisson’s ratio. Christensen^32^ suggested that the Vp/Vs ratio at 600 MPa varies from 1.77 for unaltered peridotite to 2.15 for pure low-temperature serpentinite. It has been also observed that minerals, which exhibit Vp/Vs ratios higher than 1.8 include plagioclase, amphibole, pyroxene, and Fe-olivine. Further, Christensen^32^ has shown that Fe substitution for Mg in pyroxene and olivine also increases the Vp/Vs ratio, thus, basic compositions are expected to be resulting in the higher Vp/Vs ratios. Note that felsic rocks having intermediate-to-high silica content could result in low Vp (< 6.7 km/s) and a low Vp/Vs (< 1.78) while anorthosite rocks having high plagioclase content have shown to result in relatively low Vp (between 6.6 and 7.1 km/s) and a high Vp/Vs (> 1.85). But, mafic rocks having low silica content could yield results in high Vp (> 6.7 km/s) and high Vp/Vs ratios (up to 1.86). Higher Vp/Vs exceeding 1.86 could be possible in the presence of high pressure fluids / melts in the subduction zones^33, 34^. In 2008, Matsubara et al.^33^ proposed that the high Vp/Vs values (~ 1.8–1.83) associated with the mantle wedge below the southwestern Japan could be due to the presence of high pressure fluids. The high Vp/Vs ratio (~ 1.85) in Costa-rica has been interpreted as a result of serpentinization (due to the presence of antogorite serpentinite and fluid/melt)^34^. Note that serpentinite layers occur in close association with eclogites in eastern Ladakh, northwest Himalaya, where serpentinites are proposed to be formed by hydration of the mantle wedge as a result of dewatering of the subducted slab^35^. Therefore, the modelled high average crustal Vp/Vs values (~ 1.85–2.13) in the Uttarakhand region might have been contributed by the presence of high-pressure fluids (probably metamorphic fluids) within the mid-crustal MHT^7, 22, 36^. Additionally, the serpentinization^19, 35, 37–40^ of the lower crust (> 20 km depth) might also contribute to the higher average crustal Vp/Vs ratio in the region. Both these sources of high crustal Vp/Vs values are caused by the continent–continent Himalayan collision process between the Indian and Eurasian plates.

From Table 1, we note that mean Moho depths, Vp/Vs value and Poisson’s ratio are found to be 40.17 ± 7.37 km, 1.81 ± 0.16, and 0.27 ± 0.06 in the outer Lesser Himalaya (OLH) while they are found to be 37.19 ± 5.84 km, 1.87 ± 0.13, and 0.29 ± 0.04 in the inner Lesser Himalaya (ILH) and 41.98 ± 4.47 km, 1.76 ± 0.09, and 0.26 ± 0.03 in MCT zone, respectively. From the above discussion, we can infer that inner lesser Himalaya is characterized by a relatively thin and highly mafic crust while a relatively thicker felsic crust characterizes the MCT zone, suggesting a north dipping crustal structure underlying the Uttarakhand Himalaya. However, the outer Lesser Himalaya is characterized by a ~ 40 km thick relatively less mafic crust. We also observe that the maximum change in mean Moho depths (zone-A as shown in Fig. 5b) occurs between ILH and MCT zone, where most of the micro-earthquakes including the 1991 M_w_6.6 Uttarkashi and 1999 M_w_6.4 Chamoli earthquakes have occurred.

Our modelling results reveal that areas (within the zone-A as shown in Fig. 5b) in the Uttarakhand Himalaya with marked changes in Moho depths and crustal composition below the MCT zone plausibly could be the locales of future moderate to large earthquakes. Our modelling identifies a marked change in dip angles of the Moho boundaries below the region between ILH and MCT zone (Fig. 5a,b). Thus, the increase in bending of Indian plate below the MCT zone (i.e. the region between just south of MT and VT) of the Uttarakhand Himalaya might be resulting in large concentration of seismicity in the region. Conrad and Hager^40^ suggested that the bending portion of the subducting slab can account for the ~ 60% of energy dissipation. We notice that the large earthquakes like 1991 UK and 1999 CH events took place in the bending portion of the Moho of the Indian plate (Figs. 4 and 5). Our results suggest that the thinning of Moho beyond MT in Uttarakhand Himalaya represents the bending of the Indian plate. Note that similar tectonic situation for the occurrences of mega-earthquakes have also been reported from the Sikkim-Darjeeling Himalaya^41, 42^. Several modelling studies have shown that most (~ 90%) of the elastic bending stress is released through the occurrences of earthquakes e.g. the 2004 off Sumatra mega earthquake^42^.

The recent GPS study in the Uttarakhand Himalaya^13^ suggested a long-term convergence rate of 18 mm/yr, which can result in an accumulation of 9 m slip on the MHT in 500 yrs, resulting in M ≥ 8 earthquakes in the Kumaon – Garhwal Himalaya. In support of this prediction, Vorobieva et al.^3^, based on results of the block-and-fault dynamics modelling of the Himalayan frontal arc, have also suggested that the western Nepal and Kumaon–Garhwal Himalayan regions are the most probable zones for future large to great earthquakes. Further, Yadav et al.^13^ showed a strong coupling of MHT below the lesser Himalaya in the Uttarakhand region, which along with a strain rate of 14 mm/yr for last 500 years makes this region highly vulnerable region. Thus, our modelling results would be very important to reduce the hazard associated with the most hazardous region of Himalaya.

## Conclusions

The 3-D spatial distributions of Moho depths and average crustal Vp/Vs values have been modelled, through the H–K stacking of radial PRFs at 42 broadband stations in the Kumaon–Garhwal Himalayan region. The main finding of our study is the marked lateral variations in the thickness and Vp/Vs values of the crust over the whole region. The minimum crustal thickness of 28.3 km is modelled at BTL station in Kumaon Himalaya while the maximum crustal thickness of 52.9 km is obtained at PAUR station in Garhwal Himalaya. The average [H, k] values are modelled to be [(40.17 ± 7.37) km, (1.81 ± 0.16)], [(37.19 ± 5.84) km, (1.87 ± 0.13)] and [(41.98 ± 4.47) km, (1.76 ± 0.09)] in OLH, ILH and MCT zone, respectively. Our estimates are in good agreement with the crustal thickness estimates from other studies in the region. We detect three zones (viz., R_1_, R_2_ and R_3_) with marked thinning of mafic crust, which are interspaced by zones of thickened felsic crust. These zones bend toward northeast below the MCT zone and Greater Himalaya, representing the northward subducted Indian plate (Fig. 5). The R1 and R2 zones correlate well with the spatial locations of the northeast ward extension of the DHR and MF, respectively. The NE-ward extensions of R1 and R2 below the MCT zone also coincide well with the epicentral locations of the 1991 M_w_6.8 Uttarkashi and 1999 M_w_6.4 Chamoli earthquakes, respectively (Fig. 4a,b). Our modelling also detects a zone (i.e. Zone-A as shown in Fig. 5b) of marked changes in Moho depths and crustal composition below the MCT zone and the region just north of it, suggesting the northeast-ward bending of Indian plate. The epicentral locations of most of the micro-earthquakes including the 1991 UK and 1999 CH earthquakes have occurred within the zone-A, on a north-easterly dipping plane, which might be accumulating large stresses (including plate bending stresses) due to the continued northward convergence of the Indian plate resulting in the generation of earthquakes on the MHT (high pressure fluid filled zone) below the MCT zone. Some prominent geological features like Almora Klippe, Lansdowne Klippe and Alkananda basin are noticed to be associated with the crustal thickening while the Kroll nappe is found to be associated with marked Moho up-warping. Based on our modelling results, we predict that the zones with marked thinning of mafic crust (bending of the Indian plate) below the MCT zone could be representing possible nucleation zones for future moderate to large earthquakes in Uttarakhand Himalaya.

## Methods

### Estimation and H–K Stacking of P-radial receiver functions

Amplitude and time of P-to-S conversions (Ps) from the Moho and reverberations associated with interfaces below a recording site are generally modelled by receiver function method (Fig. S1). The direct P-waves are generally impulsive on the vertical component seismogram at epicentral distances exceeding 30° while Ps conversions dominate the horizontal components of ground-motion (Fig. S1). The amplitude of conversion (Ps) and multiples (PpPms, PpSms, etc.) depends on the incidence angle of impinging P-waves and size of the velocity contrasts at the interfaces. While P-wave incidence angle and ray parameter control the arrival times of conversions and multiples on the radial RF. All possible P-to-S conversions and multiples beneath a seismic station are shown in Figs. 2a–l, S1, S2a-o, and S3a-j.

To illustrate details of the receiver function analysis procedure, we discuss here the analysis of broadband data of 3000 three-component waveforms of 1400 good teleseismic earthquakes from 42 broadband stations (Fig. 1a,c). First, we study a total of 1400 individual radial RFs from all 42 stations as a function of horizontal slowness ranging from 0.047 to 0.077 s/km and back azimuth varying from 38° to 309°. In this study, a minimum of 21 and a maximum of 35 individual radial RFs at 42 different stations are used to estimate the Moho depths through H–K stacking^16^ of PRFs. Before stacking, a moveout correction is applied using the modified IASP91 global reference model^43^ and a reference slowness of 6.4 s/° permitting summation of records from different distances. For the H–K stacking, we selected normal move-out corrected individual radial RFs (for different ranges of azimuths and epicentral distances for different stations). It is quite clear that the individual radial RFs show clear and sharp P-to-S conversions (Pms at 3.6–7.0 s after Pp (i.e., direct P arrival)) and some weak multiples (i.e., PpPms and PpSms + PsPms) from the crust–mantle boundary, suggesting that there is probably a clear Moho underlying the study region (Fig. 2a–l, see Supplementary Figs. S2a-o, S3a-j). We notice that prominent Pms arrivals characterise all individual radial RFs from all 42 broadband stations (Fig. 2a–l, see Supplementary Figs. S2a-o, S3a-j), while some weak Moho multiples are also noticed in all individual radial RFs. Finally, the crustal Vp/Vs and Moho depths have been estimated at 42 stations through the H–K stacking^16^ of moveout corrected radial P-RFs over the available range of horizontal slowness and back-azimuths (Table 1).

Zhu and Kanamori^16^ outlined a simple method to estimate the crustal thickness and the average crustal Vp/Vs ratio. They used a migration scheme for the direct Ps conversions and the crustal multiples for a set of receiver functions, assuming crustal homogeneity. They defined a quantity S(H,k) representing the weighted sum of the receiver function amplitudes at the calculated times of predicted arrivals of Ps, PpPms and PpSms + PsPms, which is expected to be maximum for a correct combination of H and k. This quantity can be written as:

1 $${\text{S}}\left( {{\text{H}},{\text{ k}}} \right)~{\text{w}}_{{\text{1}}} {\text{r}}_{{\text{j}}} \left( {{\text{t}}_{{\text{1}}} } \right){\text{ }} + {\text{ w}}_{{\text{2}}} {\text{r}}_{{\text{j}}} \left( {{\text{t}}_{{\text{2}}} } \right){\text{ }}{-}{\text{ w}}_{{\text{3}}} {\text{r}}_{{\text{j}}} \left( {{\text{t}}_{{\text{3}}} } \right)]$$

where w_1_, w_2_, and w_3_ are weighting factors, which are chosen to balance the contribution from three phases viz., Ps, PpPms and PpSms + PsPms. And, r_j_(t) is the amplitude of the receiver function for the j^th^ component while t_1_, t_2_, and t_3_ are predicted arrival times of Ps, PpPms and PpSms + PsPms phases.

The moveout times for the respective phases are given by the following formulas:

2 $${\text{t}}_{{{\text{Ps}}}} = {\text{h }}\left( {{\text{a}} - {\text{b}}} \right),$$

3 $${\text{t}}_{{{\text{Pp}} + {\text{Ps}}}} = {\text{ h }}\left( {{\text{a}} + {\text{b}}} \right)$$

4 $${\text{t}}_{{{\text{Pp}} + {\text{Ss}}}} {\text{ = h2a}}$$

where h denotes the crustal thickness, and a and b are defined as:

5 $${\text{a }} = {\text{ }}\left( {{\text{1}}/{\text{Vs}}^{{\text{2}}} {-}{\text{ p}}^{{\text{2}}} } \right)^{{{\text{1}}/{\text{2}}}}$$

6 $${\text{b}} = \left( {{\text{1}}/{\text{Vp}}^{{\text{2}}} {-}{\text{ p}}^{{\text{2}}} } \right)^{{{\text{1}}/{\text{2}}}}$$

and p is the horizontal ray parameter.

Further, we model Poisson’s ratio from the estimated Vp/Vs values using the following relation:

7 $$\sigma = \frac{{\left[ {1 - 2\left( {\frac{{Vs}}{{Vp}}} \right)^{2} } \right]}}{{2\left[ {1 - \left( {\frac{{Vs}}{{Vp}}} \right)^{2} } \right]}}$$

## Supplementary Information

Supplementary Information.
