# Supplementary material for: Modelling of crustal composition and Moho depths and their Implications toward seismogenesis in the Kumaon–Garhwal Himalaya
Source: Sci Rep. 2021 Jul 7;11:14067. doi: 10.1038/s41598-021-93469-1 (PMC8263567; doi:10.1038/s41598-021-93469-1)
Supplement: Supplementary file 1 — Supplementary Information. [file 41598_2021_93469_MOESM1_ESM.docx]

Supplemental Information

**Manuscript title**: Modelling of crustal composition and Moho depths and their Implications toward seismogenesis in the Kumaon-Garhwal Himalaya

**Authors**: Prantik Mandal, D. Srinivas, G. Suresh, and D. Srinagesh

This material includes a description of data and algorithms. It also presents the results of the HK stacking^1^ of radial PRFs. It includes 5 figures showing radial PRFs and results of HK stacking of radial PRFs.

Data and Seismic Network

In 2017, a seismic network consisting of fifty-six 3-component broadband seismographs was installed by CSIR-NGRI, Hyderabad, in the Uttarakhand Himalayan region (Fig.1a & 2a). Each station is equipped with a 24-bit Reftek-130 recorder, 120s Reftek 3-component broadband sensor and a GPS clock for time tagging. The data is recorded at 100 samples/s. The seismographs were located on hard rock sites for achieving higher signal to noise ratio. During February 2017 - February 2020, 1400 good teleseismic earthquakes were recorded by the 42 seismographs from the above network (Fig. 1c). For the present study, we used the above-mentioned dataset to compute radial PRFs through time domain deconvolution method of Ligorria and Ammon^2^.

H-K stacking of radial PRFs

Here, we use 1500 radial P-receiver functions showing clear P-to-s conversions associated with the Moho and other crustal multiples (Fig. S1), which are estimated through the time domain deconvolution (with a Gaussian width=2.5) procedure of Ligorria and Ammon^2^ with 200 iterations, from 42 broadband stations in the Uttarakhand region (Fig.1a), to conduct the H-K stacking of P-RFs (Figs. 2a-l, see Supplementary Figs. S1-5). Due to the uneven azimuthal distribution of teleseismic events (Fig. 1b), reliable anisotropic structures are difficult to retrieve with this dataset, and so we invert only for isotropic crustal structure. For HK stacking of P-receiver functions^1^, we used radial PRFs from 42 stations to delineate Moho depths and average crustal Vp/Vs values in the Uttrakhand Himalayan region.

References:

1. Zhu, L. and Kanamori, H. (2000) Moho Depth Variation in Southern California from Teleseismic Receiver Functions. Journal of Geophysical Research, 105, 2969-2980. http://dx.doi.org/10.1029/1999JB900322
2. Ligorria, J.P., Ammon, C.J., 1999. Iterative deconvolution and receiver-function estimation. Bull. Seismol. Soc. Am. 89(5), 1395–1400.

Supplementary information of 5 figures:


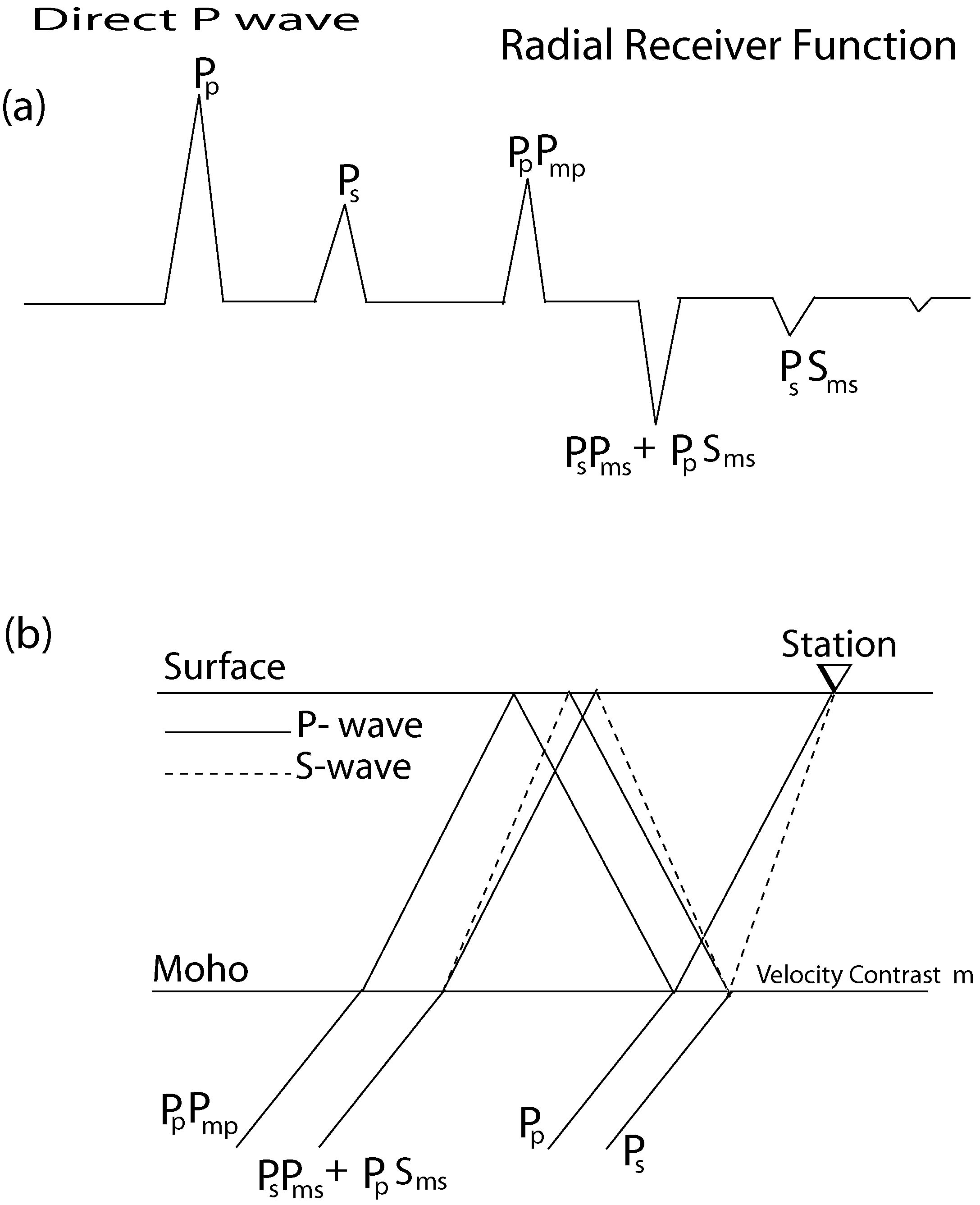


Figure S1: (a) A radial P-receiver function showing arrivals of various direct and converted phases of teleseismic P phases and (b) A plot showing raypaths of P-to-s conversions from the Moho and other crustal multiples.


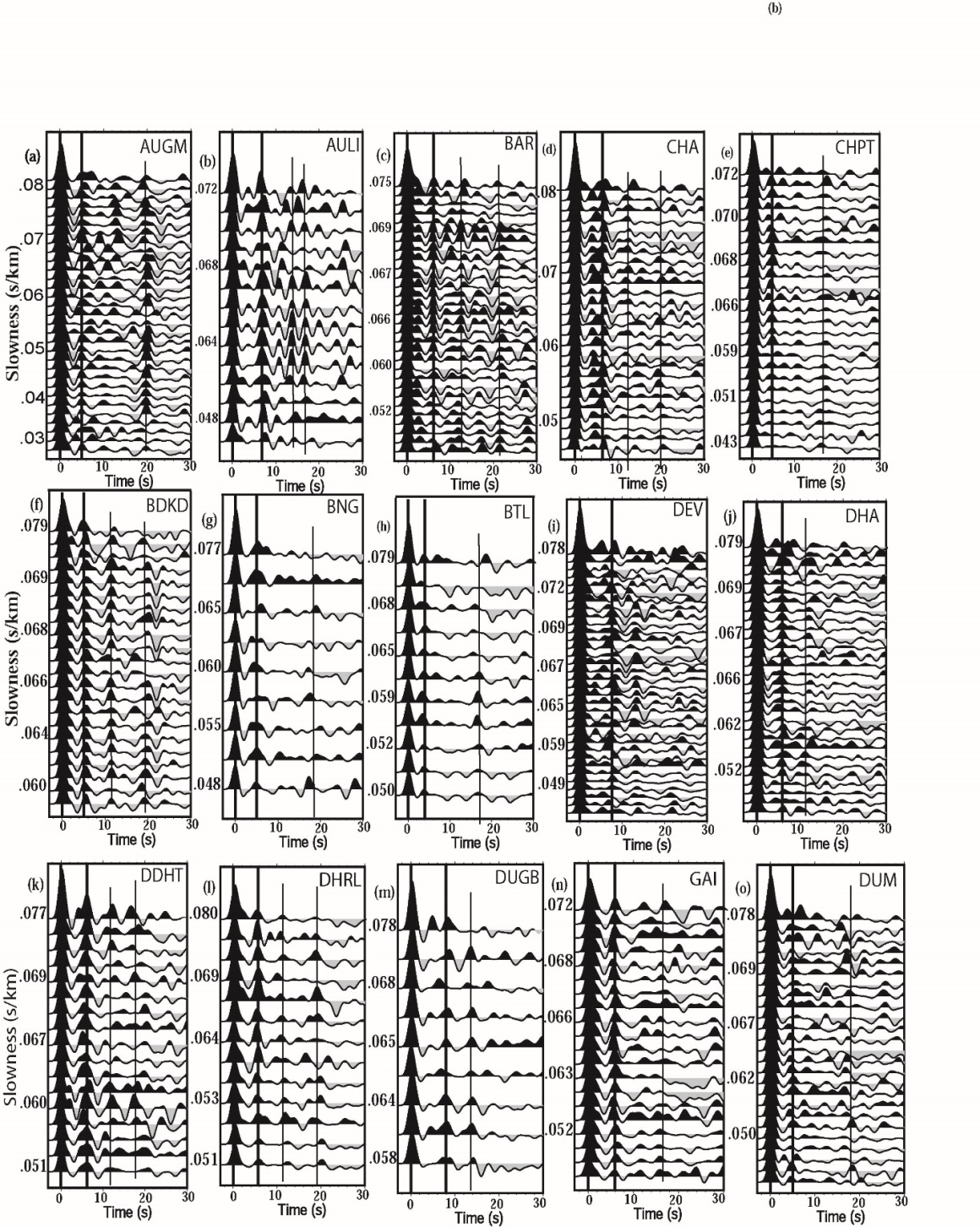


Fig S2: Plots of individual RFs as a function of the horizontal slowness after distance moveout correction for the *Ps* phase to a reference distance of 67^◦^ and slowness 6.4 s deg−1, for 15 broadband sites in the UK Himalaya, (a) AUGM, (b) AULI, (c) BAR, (d) CHA, (e) CHPT, (f) BDKD, (g) BNG, (h) BTL, (i) DEV, (j) DHA, (k) DDHT, (l) DHRL, (m) DUGB, (n) GAI, and (o) DUM. The PRFs at each station show strong azimuthal variation. The conversions from Moho and crustal multiples are marked by solid black lines.


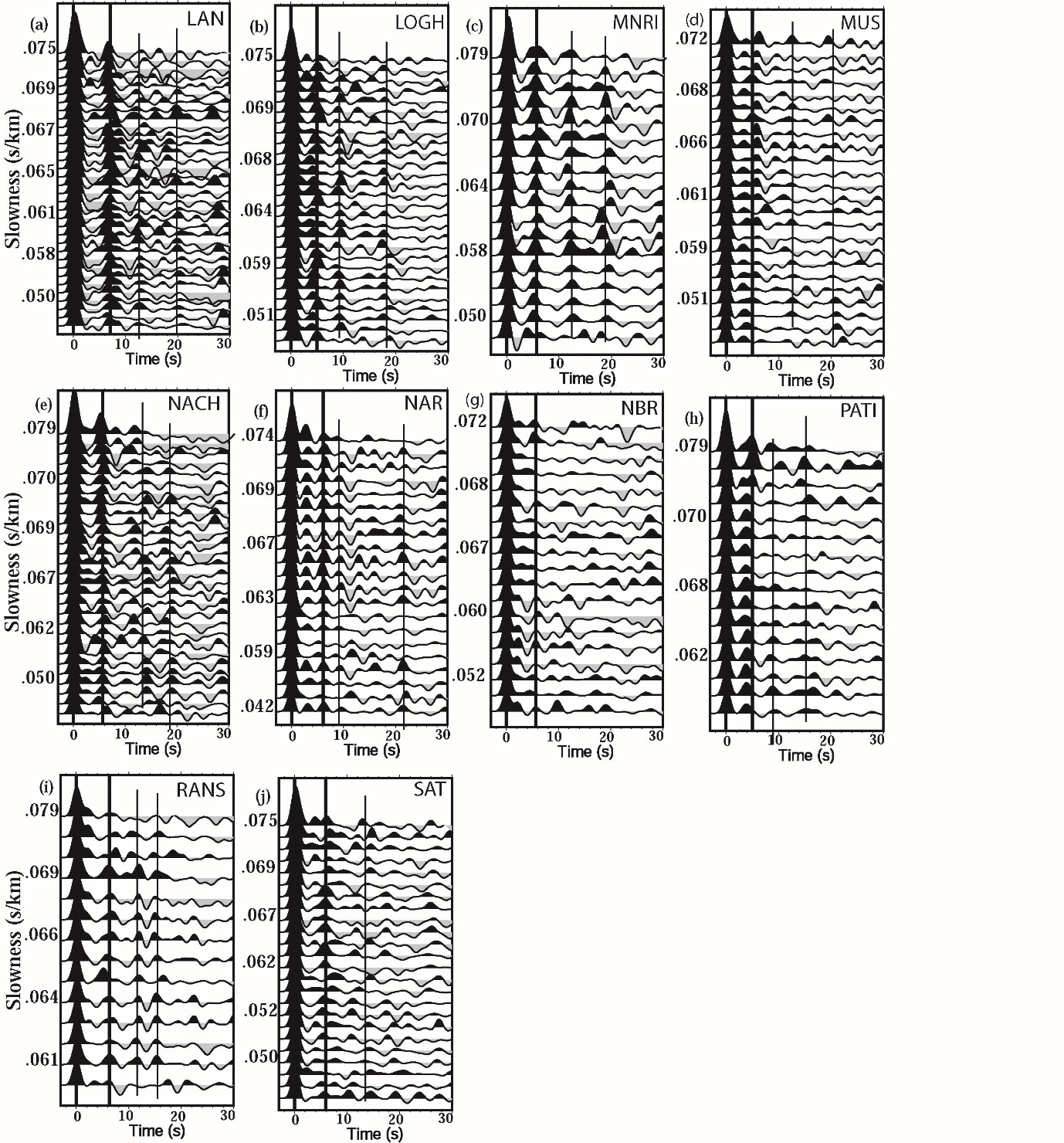


Fig S3: Plots of individual RFs as a function of the horizontal slowness after distance moveout correction for the *Ps* phase to a reference distance of 67^◦^ and slowness 6.4 s deg^−1^, for 10 broadband sites in the UK Himalaya, (a) LAN, (b) LOGH, (c) MNRI, (d) MUS, (e) NACH, (f) NAR, (g) NBR, (h) PATI, (i) RANS, and (j) SAT. The PRFs at each station show strong azimuthal variation. The conversions from Moho and crustal multiples are marked by solid black lines.


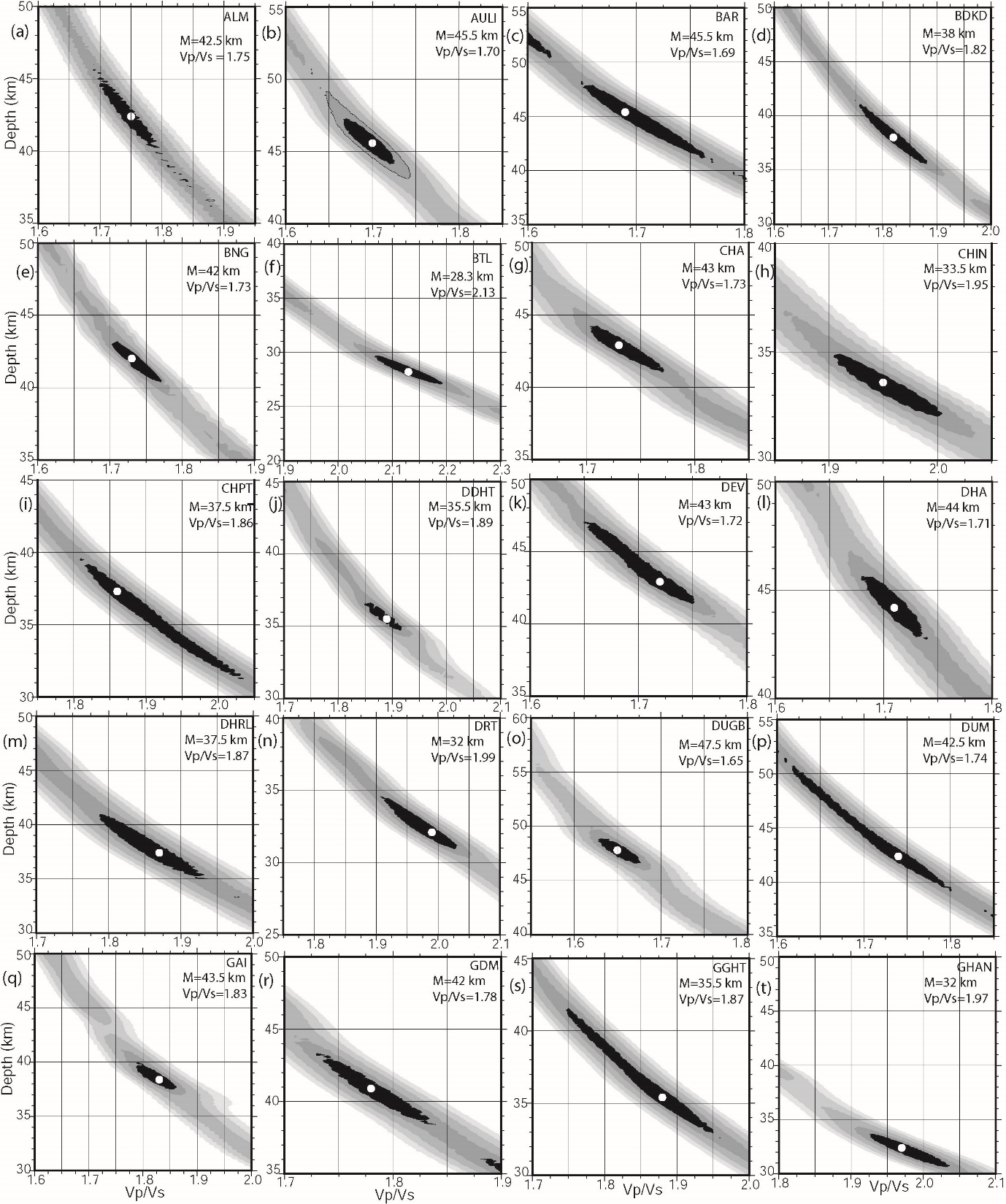


Fig S4: H-K Stacking of PRFs at 20 broadband seismograph sites in the UK Himalaya: (a) ALM, (b) AULI, (c) BAR, (d) BDKD, (e) BNG, (f) BTL, (g) CHA, (h) CHIN, (i) CHPT, (j) DDHT, (k) DEV, (l) DHA, (m) DHRL, (n) DRT, (o) DUGB, (p) DUM, (q) GAI, (r) GDM, (s) GGHT, and (t) GHAN. The best estimated H and K values are indicated by a small white filled circle at the centre of the black error ellipse.


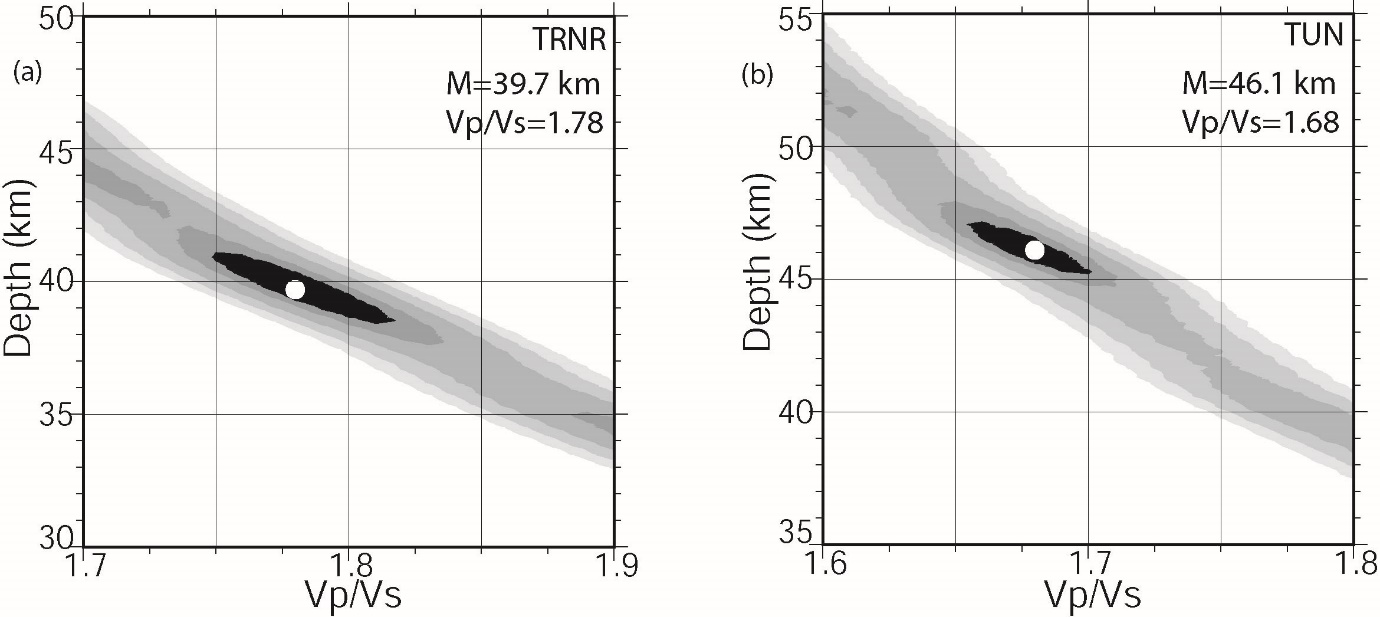


Fig S5: H-K Stacking of PRFs at 20 broadband seismograph sites in the UK Himalaya: (a) TRNR, and (b) TUN. The best estimated H and K values are indicated by a small white filled circle at the centre of the black error ellipse.
